# Artificial Intelligence and Its Role in the Management of Chronic Medical Conditions: A Systematic Review

**DOI:** 10.7759/cureus.46066

**Published:** 2023-09-27

**Authors:** Sanjana Singareddy, Vijay Prabhu SN, Arturo P Jaramillo, Mohamed Yasir, Nandhini Iyer, Sally Hussein, Tuheen Sankar Nath

**Affiliations:** 1 Internal Medicine, California Institute of Behavioral Neurosciences & Psychology, Fairfield, USA; 2 General Practice, California Institute of Behavioral Neurosciences & Psychology, Fairfield, USA; 3 Research, California Institute of Behavioral Neurosciences & Psychology, Fairfield, USA; 4 Surgical Oncology, California Institute of Behavioral Neurosciences & Psychology, Fairfield, USA

**Keywords:** ai and machine learning, ehealth technologies, comprehensive primary health care, chronic medical conditions, artificial intelligence in medicine

## Abstract

Due to the increased burden of chronic medical conditions in recent years, artificial intelligence (AI) is suggested in the medical field to optimize health care. Physicians could implement these automated problem-solving tools for their benefit, reducing their workload, assisting in diagnostics, and supporting clinical decision-making. These tools are being considered for future medical assistance in real life. A literature review was performed to assess the impact of AI on the patient population with chronic medical conditions, using standardized guidelines. A MeSH strategy was created, and the database was searched for appropriate studies using specific inclusion and exclusion criteria. The online database yielded 93 results from various databases, of which 10 moderate to high-quality studies were selected to be included in our systematic review after removing the duplicates, screening titles, and articles. Of the 10 studies, nine recommended using AI after considering the potential limitations such as privacy protection, medicolegal implications, and psychosocial aspects. Due to its non-fatigable nature, AI was found to be of immense help in image recognition. It was also found to be valuable in various disciplines related to administration, physician burden, and patient adherence. The newer technologies of Chatbots and eHealth applications are of great help when used safely and effectively after proper patient education.

After a careful review conducted by our team members, it is safe to conclude that implementing AI in daily clinical practice could potentiate the cognitive ability of physicians and decrease the workload through various automated technologies such as image recognition, speech recognition, and voice recognition due to its unmatchable speed and non-fatigable nature when compared to clinicians. Despite its vast benefits to the medical field, a few limitations could hinder its effective implementation into real-life practice, which requires enormous research and strict regulations to support its role as a physician's aid. However, AI should only be used as a medical support system, in order to improve the primary outcomes such as reducing waiting time, healthcare costs, and workload. AI should not be meant to replace physicians.

## Introduction and background

"Predicting the future isn't magic, it's artificial intelligence." - Dave Waters. The first paper was published in Medline using the MeSH term "ARTIFICIAL INTELLIGENCE" by Fletcher to describe a tortoise robot, "MATTER WITH MINDS; a neurological research robot," a seminal paper in 1951 [1]. The burden of chronic health conditions has increased worldwide, which reduces the quality of life and increases economic healthcare expenses through increased hospitalizations with increased treatment expenditures affecting over 50% of adults in 2016, accounting for 86% of healthcare expenditure [2-4]. Physicians spend only 25% of their time on direct care of patients, whereas 31% on documentation of work, consuming most of the time for activities that do not require direct patient care, increasing the wait time of patients who require frequent visits to health care due to chronic medical conditions [5].

Recent developments in artificial intelligence (AI) can be widely used in the medical field to optimize the care of patients with chronic medical conditions. The applications could suggest precision therapy for complex illnesses and reduce medical errors [6,7]. AI is software that can "autonomously generate new constructs and knowledge structures" [3,8]. It is an algorithm that could artificially mimic human cognitive and behavioral thought processes, enabling machines to solve problems with knowledge [3,6]. There are two types of AI - "Expert Systems" and "Machine Learning." Expert Systems generate a supervised prediction that outperforms human experts in decision-making with two interdependent subsystems of a Knowledge Base (accumulated knowledge) and the Inference Engine (a reasoning system) supplementing new knowledge. In comparison, machine learning is the core of AI, requiring vast amounts of data for training [7,9].

Currently, there are several reviews on the role of AI in specific fields of neurosurgery, Alzheimer's disease, clinical diagnosis of acute and chronic conditions like acute appendicitis, ability to detect malignant cells with higher diagnostic accuracy, prediction of breast cancer recurrence, and assistance of AI in hospital management systems to minimize logistics associated monetary and temporal expenses on a larger scale [7,10-15]. AI was proven to be most remarkable in the "image recognition-related fields," where they meet the diagnostic performance of medical experts, reducing the cognitive burden on physicians and thereby increasing their efficacy [7,16,17].

Most studies revealed that the achievements of AI are comparable to that of human experts, with a high accuracy of 90-100% in predefined diagnostic decisions compared with specialty doctors and outperforming the average level of clinicians in most clinical situations except for treatment suggestions [7,16]. The performance indices of weighted errors, false positivity rates, sensitivity, and specificity were at par with expert clinicians [7]. Recent advances in AI were made in the field of precise treatment algorithms for cardiovascular diseases, home AI systems for patients with insulin abnormalities and swallowing problems by use of swallow sounds through smartphone-based real-time assessment, dermatologic level of classifying skin tumors, and appearance-based diagnoses of skin diseases, digital dermoscopy, and interpretation of intrapartum fetal heart rate [7,10,16-19].

Most current literature on AI-based conversational studies was based on experimental studies with the Chatbot prototype, which imitates a natural conversation with human users. In future research, there will be a greater possibility of evidence-based evaluation of AI-based conversational agents, The Chatbot, developed for specific chronic conditions and their impact on target patients [3]. We conducted this current systematic review to assess the future role of AI in chronic conditions and its ability to accurately diagnose based on clinical symptoms and manage the condition with minimal participation of human intelligence. The aim was to improve the quality of life for patients, while simultaneously reducing the cognitive burden on physicians.

## Review

Methods and search strategy

We used the Preferred Reporting Items for Systematic Reviews and Meta-Analysis (PRISMA) guidelines for this systematic review [20]. We reviewed the literature on how AI impacts the management of patients with chronic conditions. We initially searched using the terms Artificial Intelligence (AI), chronic medical conditions, and lifestyle diseases. The search was primarily carried out in the databases PubMed, PubMed Central, and MEDLINE. Our complete search strategy includes the following:

1. ( "Artificial Intelligence/ethics"[Mesh] OR "Artificial Intelligence/standards"[Mesh] OR "Artificial Intelligence/statistics and numerical data"[Mesh] OR "Artificial Intelligence/trends"[Mesh] )

2. ( "Multiple Chronic Conditions/drug therapy"[Majr] OR "Multiple Chronic Conditions/economics"[Majr] OR "Multiple Chronic Conditions/prevention and control"[Majr] OR "Multiple Chronic Conditions/therapy"[Majr] )

3. ( "Comprehensive Health Care/methods"[Mesh] OR "Comprehensive Health Care/organization and administration"[Mesh] OR "Comprehensive Health Care/standards"[Mesh] OR "Comprehensive Health Care/statistics and numerical data"[Mesh] OR "Comprehensive Health Care/trends"[Mesh] ) and numerical data"[Mesh] OR "Comprehensive Health Care/trends"[Mesh] )

**Figure 1:**
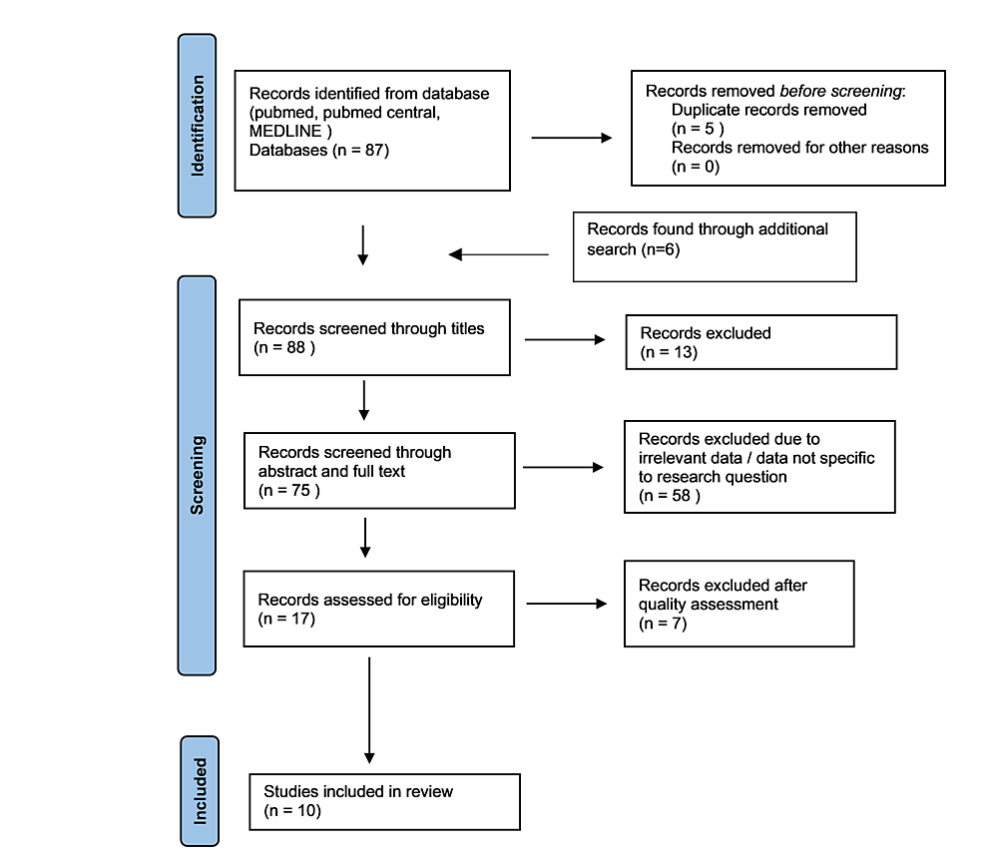
The Preferred Reporting Items for Systematic Review and Meta-Analysis (PRISMA) Flowchart

The search strategy was combined with an AND/OR. Papers provided by advanced search results were transferred to EndNote (Clarivate, Philadelphia, PA) and were exported to Excel to delete the duplicates. The titles of all the results were screened for irrelevant content and excluded accordingly. Two authors independently reviewed the abstract and full text of the remaining papers and selected the articles that satisfied the inclusion and exclusion criteria. The remaining articles were subjected to quality check and finalized for inclusion in our study (Figure 1).

Inclusion and Exclusion Criteria

We included all the articles in English and different types of articles like randomized control trials (RCTs), observational studies, systematic reviews, cross-sectional studies, and longitudinal studies. We included articles only relevant to the research question in the past ten years. We excluded editorials, peer reviews, and perspectives (Table 1).

**Table 1:** Inclusion and Exclusion Criteria

Inclusion criteria	Exclusion criteria
Articles in English language	Articles in languages other than English
Articles published <10 years ago	Articles published >10 years ago
Articles focused on research question	Articles unrelated/ only slightly related to research question
Studies other than those excluded	Peer reviews, editorials, perspectives, and unauthorized manuscripts

Analysis of Study Quality/Bias

We conducted a quality assessment on 17 papers using standardized quality assessment tools and included 10 papers that are categorized as medium or high quality in the review. We used the following tools for assessment: (1) Assessment of Multiple Systematic Review (AMSTAR) checklist for systematic reviews and meta-analysis (Table 2), (2) Scale for the Assessment of Narrative Review Articles (SANRA) checklist for traditional reviews (Table 3).

**Table 2:** Assessing the Methodological Quality of Systematic Reviews (AMSTAR) Checklist

AMSTAR Criteria	G. Damiani et al. (2023) [21]	S.A. Rahami et al. (2021) [22]	Lam TYT et al. (2022) [23]	Shen J et al. (2019) [7]	Yin J et al. (2021) [24]	A. d’Elia et al. (2022) [25]	Schachner T et al. (2020) [3]
Priori design provided	Yes	Yes	Yes	Yes	Yes	Yes	Yes
Duplicate study selection and data extraction done	Yes	Yes	Yes	Yes	Yes	Yes	Yes
Was comprehensive literature search performed	Yes	Yes	Yes	Yes	Yes	Yes	Yes
Was status of publication used as inclusion criteria	Yes	Yes	Yes	Yes	Yes	Yes	Yes
A list of inclusion and exclusion studies provided	Yes	Yes	Yes	Yes	Yes	Yes	Yes
Characteristics of inclusion studies provided	Yes	Yes	Yes	Yes	Yes	Yes	Yes
Quality of inclusion studies included and documented	Yes	No	Yes	Yes	Yes	No	No
Quality of inclusion studies used appropriately in forming conclusions	Yes	No	Yes	Yes	Yes	No	No
Appropriate methods used to combine studies	Yes	Yes	Yes	Yes	Yes	Yes	Yes
Likelihood of publication bias assessed	No	Yes	Yes	Yes	Yes	Yes	Yes
Conflict of interest declared	No	No	No	No	No	No	No
Assigned Score	9/11	8/11	10/11	10/11	10/11	8/11	8/11

**Table 3:** A Scale for the Assessment of Non-Systematic Review Articles (SANRA) Checklist

SANRA checklist	S. Reddy et al. (2019) [26]	M. J. Kasteleyn et al. (2022) [27]	J. K. Kueper et al. (2019) [28]
Justification of Article Importance	2	2	2
Concrete Aims/Formulation of Question	2	2	2
Description of Literature Search	0	0	0
Referencing of key statements	2	2	2
Scientific Reasoning/Appropriate Evidence	2	2	1
Appropriate Presentation of Data	1	2	2
Assigned Score	9/12	10/12	9/12

Results

The preliminary search yielded 20 articles through the PubMed MeSH strategy, and an additional 67 articles were retrieved through an advanced search in PubMed. Furthermore, six articles were found through different online databases, resulting in 93 studies. Two authors independently carried out the selection process. After transferring to EndNote Basic, five duplicates were removed. Subsequently, the titles of 88 articles were screened, leading to the removal of 13 studies. The remaining 75 articles were exported to an Excel spreadsheet to screen abstracts and full texts. After excluding 58 studies, 17 were selected for quality checks using standard checklists specific to the respective study type. Ten studies with moderate to high quality were included in the systematic review [3,7,21-28].

The results include three systematic reviews and seven traditional reviews (Table 4). Nine out of 10 studies favor AI implementation in clinical practice. They considered its potential use in diagnostics, image recognition, data entry, personalized medicine, and many more. In contrast, one study demonstrated no significant increase in patient outcomes compared to standard care. Two studies promoted conversational agents or chatbots and mobile eHealth applications for at-home medical management. Three studies addressed the issue of privacy protection and data safety as a potential threat. One study assessed the potential of AI in reducing medical errors and reported a positive outcome.

**Table 4:** Characteristics of Included Studies AI: artificial intelligence

Author of study	Year of study	Publication type	Aim of the study	Conclusion
Shen J et al. [7]	2019	Systematic Review	To compare the performance of AI with Clinicians focusing on disease diagnosis	The diagnostic efficiency of AI is comparable to that of Clinicians, particularly in the field of image recognition
S. Reddy et al. [26]	2019	Traditional Review	Envisioning the future of AI-enabled healthcare	AI implementation has a huge cost-saving potential and augments quality healthcare delivery
Schachner T et al. [3]	2020	Systematic Review	Utility of conversational agents (or Chatbot) in the setting of chronic medical condition	Employing conversational agents are shown to improve patient outcome with high patient engagement
Yin J et al. [24]	2021	Systematic Review	Applications of AI in real-life clinical practice	AI has a wide range of utility in clinical practice ranging from disease diagnosis to improved patient outcome and as an administrative aid
S.A. Rahami et al. [22]	2021	Systematic Review	Evaluation of implemented AI at the level of community-based healthcare	The prime use of AI lies in the field of diagnosis, detection, and surveillance of patients
J.K. Kueper et al. [28]	2021	Traditional Review	Providing education on AI-based tools to end users and Primary Care Physicians	-
M.J. Kasteleyn et al. [27]	2021	Traditional Review	Providing insight regarding the use of eHealth in primary care	-
Lam TYT et al. [23]	2022	Systematic Review	Evaluating the performance of AI-assisted tools in clinical practice	AI-assisted tools outperformed the standard clinical care and significantly improved patient outcomes
A. d'Elia et al. [25]	2022	Systematic Review	Impact of AI implementation on health inequity	AI can be safely implemented in primary care after mitigating the potential health inequities by involving the community in its implementation
G. Damiani et al. [21]	2023	Systematic Review	Comparing the outcomes of AI with that of clinicians in the field of drug management and medication errors	The efficacy of AI exceeds that of clinicians in the field of drug management and medication errors

Discussion

In the new era of modern medicine, AI is gaining tremendous popularity, with a vast amount of research for its implementation in clinical practice. AI is a science and engineering discipline devoted to the computational understanding and reproducibility of intelligent behavior in the healthcare system to reduce the burden on healthcare workers [21]. The most commonly used AI models are Machine Learning (ML), Natural Language Processing (NLP), and expert systems, whose highest performing accuracy was demonstrated by Convolutional Neuronal Networks (CNN) and abductive networks [22]. The other techniques which could be used include speech recognition, neural networks, natural language understanding, and AI markup language [3]. AI-assisted tools use Bio signals like endoscopic images or clinical data constituting blood pressure readings and blood tests to analyze data. These tools frequently rely on static data of the patient (snapshot evaluation such as an endoscopic image or a biopsy report) over dynamic data (continuous evaluation such as a Holter monitor or 24-hour ambulatory blood pressure monitor) [23].

Information technology and data mining tools are the most valuable techniques in health care administration, reducing the burden and administrative demands, increasing the efficacy of clinical care by undertaking time-consuming and repetitive tasks like the automated entry of laboratory and imaging results, patient data entry [25]. The concept of personalized medicine could be made possible through AI using patient's demands based on their profile and unique characteristics, which led to the innovative term "precision health," enhancing the quality of life through individualized treatment strategies. The computerized decision support system was the most frequently used machine category in clinical practice, significantly reducing medication and prescription errors [3]. In image recognition, computer-assisted technologies could rapidly identify the disease due to its unique non-fatigable learning skills compared to cognitive fatigue in physicians leading to higher productivity [7]. AI implementation is expected to increase patient outcomes owing to its aid in decision-making, patient-centered care, and cost reduction [24]. Currently, EndoScreener (Shanghai Wision AI, China) is the only AI-assisted tool considering ethnic diversity, using a diverse set of endoscopic images [23]. Another model of an AI-assisted tool is the conversational agent or the Chatbot, which is available for mobile users trained to interact with the patient and potentially used to collect data, diagnose or educate the patient to achieve self-care [21]. New techniques like transfer learning, voice capture with transcriptions, knowledge distillation, and injection are being developed [26].

Advantages of AI in Clinical Practice

The current study results of Shen J et al. [7] showed that the diagnostic efficacy of AI is comparable to physicians and exceeds that of physicians with low or limited experience, with high diagnostic accuracy of 90% in most clinical settings, except for the field of treatment suggestion. This performance was observed to a greater extent in image recognition. In independent studies, AI was compared to dermatologists, radiologists, and ophthalmologists, and it demonstrated higher accuracy compared to the respective physicians. No significant increase in the rate of false positives was observed when compared with the clinicians. However, the missed detection rate was lower than the ophthalmologists involved in a separate study. Lam TYT et al. [23] assessed 39 RCTs on the primary and secondary outcomes of AI-assisted interventions. Thirty out of 39 studies (73% of the interventions were based on biosignal data) showed an improved outcome compared to standard care. The extended guidelines by Standard Protocol and Items: Recommendations for Interventional Trials-Artificial Intelligence (SPIRIT-AI) state that the performance of AI-assisted tools was higher than standard care management.

A study conducted by S.A. Rahami et al. [22] with 21,325,250 patients, comparing an expert-led control group and an AI-driven treatment group, showed a significant increase in the diagnostic accuracy of the treatment group. The study showed that the benefits of AI were tremendous, including improved patient care due to increased treatment adherence, screening speed, prediction of risk factors, and others due to early diagnosis. By implementing AI, the burden on physicians was significantly reduced, enhancing the quality of care and interprofessional communication. AI can be used in various other areas, including remainder alarms for patients, issuing warnings in Electronic Medical Records (EMR), monitoring of chronic diseases, conducting clinical data analysis, and performing population-based surveillance.

Damiani G et al. [21] considered the use of AI in drug management and medication errors. The study reported a reduction in medical errors by introducing AI into physician routines in nearly 70% of the reviewed articles. Currently, healthcare professionals are already utilizing e-prescription techniques. These techniques involve computerizing the physician's order, and any dispensing errors in the prescription dosage, duplication, or potential drug interactions are instantly reported to the physician. This immediate feedback helps in reducing the occurrence of adverse effects. As there is a high prevalence of medication errors that could be easily prevented, targeting this aspect of health care through AI would dramatically reduce the harmful effects on patients.

S. Reddy et al. [26] presumed that the leading utility of AI lies in health care interventions, patient administration and monitoring, and clinical decision-making. One such use of ML was to schedule the patients and prioritize them to reduce wait times. Voice capture technology and transcription can record patient data by note-taking and documenting in EMR, allowing clinicians to spend more time with patients. The study mentioned the ability of AI in detection of septic shock, treatment of chronic diseases such as chronic obstructive pulmonary lung disease (COPD), reduction of medical errors, aiding clinical decision-making, emergent disease outbreaks surveillance, automation of 3D image analysis, acceleration of drug development, and prediction of outcomes in critically ill patients. It is also supported using Artificial Neural Networks (ANN), an ML technology, due to its unique nature of diagnosing diseases more efficiently than clinicians. It could match the performance of radiologists and pathologists. Hence, when integrated into electronic health records, ANN could reduce medical errors and suggest appropriate treatment protocols. The report mentioned that using newer mobile apps and fitness tracking devices could help track patient details such as blood pressure recording, heart tracing, and sleep patterns. AI could also be appointed to schedule patient follow-up visits and communicate medical information with patients increasing the treatment adherence and the rate of regular follow-up. The reports published by K. Keuper et al. [28] supported the above data. According to the study, AI can support multiple tasks such as predicting patient outcomes, capturing population data, supporting clinical decision-making, analyzing language data such as speech recognition, answering telephone calls, and analyzing and interpreting image data.

However, contrasting results on AI were published by Yin J et al. [24] after reviewing the implemented AI in real-life clinical practice, targeting the fields of diagnosis, treatment, triage, and risk analysis. Based on the results, an AI system called IDx-DR diagnosed diabetic retinopathy with 87.2% sensitivity and 90.7% specificity. It became the first diagnostic AI system to be approved by Food and Drug Administration (FDA), which was later found to have a high false positive rate with only a 12% positive predictive rate. Another AI-based application, CC-Cruiser (Zhongshan Ophthalmic Center, China), developed for detecting childhood cataracts, had a lower accuracy, positive predictive value (PPV) and negative predictive value (NPV). When the application assessed patient outcomes, it reported an improved outcome with reduced hospital stays, hospital mortality, intensive care unit (ICU) transfers, and readmissions. Despite the above results, the study recommended using AI in clinical decision support to improve patient outcomes and reduce healthcare costs.

Meanwhile, Schachner et al. [3] described the importance of chatbots and conversational agents for improving self-care and management through patient interaction addressing various chronic conditions. The study showed a high-performance rate with an accuracy of 89% and a precision, sensitivity, and specificity of 90%, 89.9%, and 94.4%, respectively, with a high message response rate of 81% to 97%. The research demonstrated that it improved treatment adherence and increased awareness of disease symptoms. The interaction reduced depression and anxiety in patients compared to the control population. The user experience was satisfactory, with high engagement and positive outcomes.

Kasteleyn et al. [27] proposed using eHealth applications due to their potential in reducing the clinician workload and their ability to monitor or track patients. Interventions such as at-home daily BP monitoring or blood glucose measurements compared to quarterly checkups would increase treatment adherence and provide a better patient outcome. These applications could provide patients with round-the-clock availability, increased satisfaction, and independence.

Even though AI has been proven helpful in clinical implementation, it is crucial to consider the potential for harm, reliability level, specificity and sensitivity, ethical considerations, and privacy risks through extensive research compared to the control groups.

Challenges Faced in the Application of AI

AI is a double-edged sword. The list of potential challenges equals the benefits provided by it. S.A. Rahami et al. [22] and Kasteleyn et al. [27] addressed the primary concern of privacy protection. The latter also identified a need for more transparency in the decision process made by algorithms used, requiring the implementation of strict AI regulation guidelines. Furthermore, scientific evidence of AI-assisted tools in safety and efficiency is required for the regulatory bodies to approve [23].

Nevertheless, another issue was the need for increased literacy regarding AI tools accounting for its complex nature of learning. There is a risk of bias in specific population subgroups in the setting of inappropriate training of algorithms. From the medicolegal point of view, the assignment of responsibility and liability determination in case of medical errors must be clarified and established [26].

The evidence collected by Schachner et al. [3] pointed out the misleading recommendations of AI, which could harm diagnostic accuracy. It was also stated that AI diagnostics had a high false positive rate, with a PPV of only 12% and numerous incorrect diagnoses. S.A. Rahami et al. [22] proposed that improving high-quality data for modern AI technologies is essential and necessitates strict documentation, transparency, accuracy, and robustness. Even with considerable development in AI, it can only exist with the engagement of humans, signifying its artificial nature. Another potential risk to be considered while using chatbots or conversational agents and eHealth applications includes harm or even patient death when the suggestions are inaccurate when a critical decision is involved.

In addition to the above-stated limitations, the physician's and patient's acceptance rate of AI was low. Despite the better diagnostic rate provided by AI, the patients preferred human physicians as AI could not address their unique personal needs. Moreover, the patients perceived the doctors who used the assistance of AI as less competent. Meanwhile, the physicians disliked AI implementation due to job insecurities and losing control over patient care. The lack of willingness from the care provider and receiver is a significant barrier to its clinical use. AI and the additional considerations cannot appreciate the importance of ethical and ethnic backgrounds regarding age, gender, and social aspects [24,27].

We recommend further research studies to assess the cost-effectiveness, its effect on the psychosocial aspects, medicolegal implications, privacy protection, learning abilities of physicians, the willingness of patients and clinicians to accept AI, potential harms due to inaccurate suggestions, and other aspects which could determine the success of real-life implementation.

Limitations of the Study

Our review did not include RCTs or other longitudinal studies, and the selection process may not accurately represent the available data. The potential risk of bias was not considered, and this review only summarized the findings of included studies.

## Conclusions

AI has the potential to transform the future of modern medicine. Considering the results of the above-selected studies, AI-based tools could be immensely useful in the modern medical field in aiding the clinician. Its potential uses could be seen in clinical decision support, diagnosing medical conditions, image recognition, follow-up and surveillance of patients with chronic conditions, and predicting treatment outcomes. It supports the administrative field by scheduling follow-up visits, data entry, and reducing the healthcare cost and clinician burden. From the viewpoint of patients, it could help in health education through mobile apps, provide at-home monitoring of particular illnesses, create medical alerts for follow-up visits, and many more. However, certain limitations could outweigh these benefits, creating a barrier to its implementation in clinical practice. Data security and the possibility of misjudgments in clinical diagnosis are the primary limitations that must be considered. Additional limitations of implementing AI into clinical practice include its complex nature of learning, patient harm due to possible inaccurate diagnosis, liability determination in case of medicolegal errors, patient and physician acceptance rate of AI, the inability of AI to consider ethnicity, race, and other psychosocial aspects of the patient population. This indicates the need for extensive research addressing the above concerns, and to develop a safe and accurate intervention for future use.
